# Anti-inflammatory effects of an injectable copolymer of fatty acids (Ara 3000 beta®) in joint diseases

**DOI:** 10.1186/s12950-015-0062-7

**Published:** 2015-02-23

**Authors:** Catherine Baugé, Eva Lhuissier, Nicolas Girard, Céline Quesnelle, Gérard Ewert, Karim Boumediene

**Affiliations:** Normandie Univ, Caen, France; UNICAEN, EA4652 MILPAT UFR de médecine, Université de Caen, Caen cedex 5, CS14032 Caen, France; Sexmoor Laboratoires, 13 120 Saint Remy de Provence, France

**Keywords:** Cytokines, Drug therapy, Extracellular matrix, Fatty acid, Gene expression, Inflammation, Arthritis, Osteoarthritis, MMPs, Joints

## Abstract

**Background:**

In inflammatory joint disease, such as osteoarthritis or arthritis, there is an increased level of pro-inflammatory cytokines, such as interleukin-1β. These cytokines stimulate the expression and release of matrix metalloproteases (MMP), leading to the degradation of cartilage extracellular matrix and subsequently mobility difficulty and suffering for patients. The aim of this study was to examine the therapeutic potential of a fatty acid copolymer in *in vitro* and *in vivo* models of cartilage inflammation.

**Methods:**

Inflammation was mimicked *in vitro* by treatment of human articular chondrocytes with interleukin-1β. Effects of a co-treatment with a copolymer of fatty acids (Ara 3000 beta®) were determined by evaluating MMP production by RT-PCR and ELISA, NO release by Griess assay, and PGE2 expression by ELISA. In addition, *in vivo* analysis (evolution of weight and edema) were also performed after injection of Freund adjuvant in rats treated or not with the copolymer of fatty acids.

**Results:**

The copolymer of fatty acids clearly reduces inflammation in joint. *In vitro*, it impairs IL1 stimulated-MMP production and release, as well as the release of NO and PGE2 and the activation of NFκB. Furthermore, *in vivo* experiments using adjuvant induced-arthritis corroborates the anti-inflammatory effects of the copolymer of fatty acids, with a reduction of edemas, erythemas and ankylosis in arthritic rats.

**Conclusions:**

The results support the hypothesis that a copolymer of fatty acids, such as Ara 3000 beta®, is a powerful anti-inflammatory compounds, suggesting that it has a potential for preventing cartilage degradation associated with chronic inflammatory joint disease.

## Background

Joint diseases such as the degenerative joint disease osteoarthritis (OA) and the inflammatory disease rheumatoid arthritis (RA) are characterized by dramatic degradation of articular cartilage, caused by overexpression of matrix metalloproteinases (MMPs), and reduced anabolic activity of chondrocytes. The MMPs, produced by chondrocytes, are zinc-containing proteinases that degrade cartilage and cause severe joint damage. MMP-1 and MMP-13, particularly cause the digestion of type II collagen, the principal collagenous material in hyaline cartilage. MMP-3 is also involved in the breakdown of extracellular matrix by degrading proteoglycans.

IL-1β has been detected in the synovial fluid of OA patients [1] and plays a major role in joint diseases, causing overproduction of prostaglandin E2 (PGE2), reactive oxygen species (ROS), nitric oxide (NO), and MMPs, which all contribute to the degradative process of cartilage. IL-1β also reduces levels of inhibitors of MMPs (tissue inhibitors of MMPs, TIMP) in arthritic joints and decreases biosynthesis of type II collagen and aggrecan, limiting the repair potential of cartilage. These effects have been confirmed by *in vivo* studies showing that intraarticular administration of IL-1β into animal joints causes proteoglycan loss from the cartilage, whereas injection of IL-1 receptor antagonist (IL-1ra) protects cartilage in arthritic joints. Regarding these data, numerous *in vitro* studies have evaluated the interest of anti-arthritic molecules using their ability to counteract IL-1 effects in models of inflammatory joint diseases. Such studies have highlighted the anti-inflammatory effects of tested drugs by measuring the repression of expression of inflammatory markers such as MMP levels, PGE2 or NO induced by IL-1 [2]. However, even if IL-1β is a crucial factor in cartilage degradation mechanisms, other proinflammatory cytokines, especially TNF-α and IL6, play also major roles and can strongly stimulate inflammation in arthritis.

The main objective of drugs used in joint diseases, such as non steroidal anti-inflammatory drugs (NSAIDs), TNF-α inhibitors, or IL-1 receptor antagonists, is symptomatic management, reducing both pain and underlying inflammation [3]. However, given the cost of such biologic agents and their limited efficacy in some patients [4,5], there has been a great demand for the development of novel therapeutic agents.

Certain polyunsaturated fatty acids are beneficial to the health of mammals. For instance, long chain n-3 fatty acids are present in fish and marine mammals, and epidemiological data indicate a correlation between fish-wealthy diets and reduced incidence of inflammatory diseases [6]. In addition, in a variety of conditions, including rheumatoid arthritis or osteoarthritis, these fatty acids have been shown to offer therapeutic activity [7-9]. This may be associated with increased collagen synthesis and decreased amounts of the inflammatory mediator prostaglandin E2 as reported in fibroblasts *in vitro* [10]. At contrary, n-6 fatty acids have been reported to induce IL-1, IL-6, TNF-α and PGE2. However, n-3 fatty acids inhibit n-6 fatty acid induced-cytokine expression [11]. More recently, it has been shown that veterinary therapeutic diet rich in omega-3 fatty acids improved the locomotor disability and the performance in activities of daily living of OA dogs [12]. A beneficial effect was also shown in OA-prone Dunkin Hartley strain guinea pigs on a diet rich in long chain omega 3 fats with an improvement of OA degree and severity [13].

Besides, a copolymer of fatty acids, composed of oleic acid, palmitic acid and stearic acid, called Ara 3000 beta® has also been shown to reduce osteoarthritis symptoms in dogs, with a reduction of lameness and pain [14]. This injectable gel also inhibits the synthesis of leukotriene and the degranulation of mast cells.

Here, we wanted to evaluate the anti-inflammatory functions of this fatty acid copolymer in joint. So, we evaluated its effects on IL-1-induced MMP production and other markers of inflammation in articular chondrocyte culture. Then, we used an *in vivo* model of arthritis to evaluate the therapeutic interest of this fatty acid copolymer against inflammatory joint diseases.

## Methods

### Reagents

Reagents were supplied by Invitrogen (Fisher Bioblock Scientific, Illkirch, France) unless otherwise noted. IL-1β (Sigma-Aldrich, St. Quentin Fallavier, France) was resuspended in phosphate buffered saline (PBS) with BSA. Oligonucleotides were supplied by Eurogentec (Angers, France). The copolymer of fatty acids Ara 3000 beta® (oleic acid: 8.75 mg/ml, palmitic acid: 5.4 mg/ml and stearic acid: 4 mg/ml) was supplied by Sexmoor Laboratory and diluted at 1:10 in Phosphate Buffer Saline (PBS) supplemented with 0.25% Bovine Serum Albumin (BSA). This solution was used at two dilutions (1:250 and 1:1000) in 10% FCS and 0.25% BSA-containing RPMI medium to reach the final concentrations (1:250 dilution corresponding to 1.28 10^−4^ M oleic acid, 1.88 10^−4^ M palmitic acid, and 2.84 10^−4^ M stearic acid; or 1:1000 dilution corresponding to 3.2 10^−5^ M oleic acid, 4.7 10^−5^ M palmitic acid, and 7,11 10^−5^ M stearic acid).

### Cell culture and treatments

Human articular chondrocytes (HAC) were prepared from femoral heads of patients with hip fractures All donors signed agreement forms before the surgery, according to local legislation (agreement obtained from local ethical committee, “Comité de Protection des Personnes Nord Ouest III”). Cells were isolated and cultured as previously described [15]. Cartilage samples were cut into small slices, and chondrocytes were isolated by sequential digestion by type XIV protease (Sigma-Aldrich, St. Quentin Fallavier, France) and then type I collagenase (from Clostridium histolyticum; Invitrogen, Fisher Bioblock Scientific, Illkirch, France). The cell suspension was filtered, seeded in plastic vessels at a density of 4 × 10^4^ cells/cm^2^ and cultured in Dulbecco’s modified Eagle’s medium (DMEM) supplemented with 10% fetal calf serum (FCS; Invitrogen) and antibiotics, in an atmosphere of 5% CO_2_. The medium was changed twice a week.

On day 8, the cells were incubated with 10% FCS and 0.25% BSA-containing RPMI medium with Ara 3000 beta® (dilution 1:1000 or 1:250). The next day, medium were changed and cells were incubated with fresh Ara 3000 beta® according the same protocol, and supplemented or not with interleukin-1β (IL-1β) (1 ng/ml; Sigma-Aldrich, St. Quentin Fallavier, France).

### Viability assay

Viability was evaluated by tetrazolium colorimetric WST1 assay (Roche Diagnostics, Meylan, France). HAC were plated into 96-well microtiter plates. At 70% confluence, Ara 3000 beta® at increasing dilutions was added in all wells, except control. Then, microplates were incubated for 48 h. One hour before the end of incubation, 10 μl of WST1 solution was added to all wells. Optical density was measured on a spectrophotometer plate reader (1420 Multilabel Counter, Perkin Elmer) at 450 nm. A well without cells containing complete medium and WST1 only acted as blanks. Survival was calculated according to the formula: OD_test_/OD_control_.

### RNA extraction and real-time reverse transcription–polymerase chain reaction

Total RNA from primary HAC cultures were extracted using Trizol. After extraction, 1 μg of DNase-I–treated RNA was reverse transcribed into cDNA in the presence of oligodT and Moloney murine leukemia virus reverse transcriptase. The reaction was carried out at 37°C for 1 h followed by a further 10-min step at 95°C. Amplification of the generated cDNA was performed by real-time PCR in an Applied Biosystems SDS7000 apparatus with appropriate primers designed with Primer Express software. The relative mRNA level was calculated with the 2^–ΔΔCT^ method.

### Elisa

PGE2 and MMPs released into conditioned media was quantified using commercially available enzyme immunoassay kit (R&D Systems, Lille, France). Absorbance was determined at 450 nm with a wavelength correction set at 540 nm.

### NO assay

Generation of nitric oxide was determined by measuring nitrite accumulation in culture supernatants using Griess reagent (1% sulphanilamide and 0.1% N-(1-naphthyl)-ethylenediamine dihydrochloride in 5% H3PO4; Sigma-Aldrich, St. Quentin Fallavier, France). Sample and Griess reagent (150 μl of each) were mixed and incubated for five minutes; the absorption was estimated by a plate reader (1420 Multilabel Counter, Perkin Elmer) at 540 nm. Sodium nitrite (NaNO2, Sigma-Aldrich, St. Quentin Fallavier, France) was used as positive control.

### Nuclear extracts and EMSA

Extracts were prepared as previously described [16]. The protein amount was determined by the Bradford colorimetric procedure (Bio-Rad, Marnes-la-Coquette, France). For electrophoretic mobility shift assay (EMSA), 5 fentomoles of biotinylated probe (corresponding to consensus NFkB binding site) and 5 μg of nuclear proteins from chondrocytes were incubated at room temperature for 30 min in binding buffer (50 mM Tris–HCl pH 8, 1 mM dithiothreitol, 750 mM KCl, 2.5 mM EDTA, 0.5% Triton-X100; 65.5% glycerol (v/v)) with poly-dIdC (1 μg). Then all samples were loaded on a nondenaturating acrylamide gel (7%). After separation, probes and proteins were electrotransferred on PVDF membrane, which was incubated with streptavidin-conjugated peroxydase (Sigma). The signals were revealed with SuperSignal West Pico Chemiluminescent Substrate (Pierce Perbio Science, Brébières, France) and exposed to X-ray film.

### In vivo experiments

Animal experimental procedures were performed in the animal facility of Valbex Center Lyon1 (Villeurbanne, France) according to local legislation. The procedure was approved by internal ethics committee of Valbex facility. The discussions addressed the ethics aspect of study and the respect of 3Rs. Researchers held a nominative authorization to animal experiment provided by the French Ministry of Agriculture. This authorization also supported the ethics of studies with animal appearance.

The animals were kept in the animal facility (Valbex Center Lyon1,Villeurbanne, France) and allowed to acclimatize to the laboratory conditions (temperature 23 ± 2°C with a 12 h light–dark cycle). Animals had free access to standard rat chow and water. Each animal was used only once as all animals were euthanized at the end of each experiment. The animals were humanely handled throughout the experiment in accordance with internationally accepted ethical principles for laboratory animal use and care, and all efforts were made to minimize animal suffering. Euthanasia was performed using carbodioxyde inhalation or barbiturate overdosis.

Adjuvant-induced arthritis (AIA) was produced on day 0 by an intradermal injection of 500 μl of a complete Freund adjuvant suspension (DIFCO, ref 0638–607) into the right hind paw of male Wistar EOPS rats under brief anesthesia with isoflurane (<3 minutes, 4% isoflurane). The development of AIA was assessed by paw volume changes and appearance of secondary lesions. The volume of the injected and noninjected hind paw was determined by plethysmography. Measurements were performed over a 30-day period. Body weight was assessed daily. Arthritis severity score was evaluated in a scale going from 0 to 4 with 0 corresponding to no erythema and no inflammation; 1: cutaneous erythema, 2: moderate inflammation (edema) and erythema; 3: severe inflammation; and 4: very severe inflammation with ankylosis. Scoring was always done by the same person in a blind way.

For experiments, the animals (n = 10 per group) were divided into 3 treatment groups: (1) animals with arthritis which received intramuscular injections of 600 μl of physiological serum one day before Freund adjuvant injection, and 300 μl of physiological serum at day 6, 9 and 12 (group control); (2) animals with arthritis which received intramuscular injection of 600 μl of Ara 3000 beta® the day before the Freund adjuvant injection and 300 μl of the copolymer of fatty acids at day 6, 9 and 12 (group Ara 3000 beta®), and (3) animals with arthritis which daily received aqueous solution of acetylsalicylic acid (200 μg/g) *per os,* from day −1 to day 12.

### Statistical analysis

For *in vitro* experiments, three different experiments were performed. The values are means ± SEM and the significance of differences was calculated with Student’s *t* test. For in vivo experiments, 10 animals were used for each group, and ANOVA tests (repeated measures) followed by Fisher PLSD test, and Student’s test were done.

## Results

### Toxicity of the fatty acid copolymer

**Figure 1:**
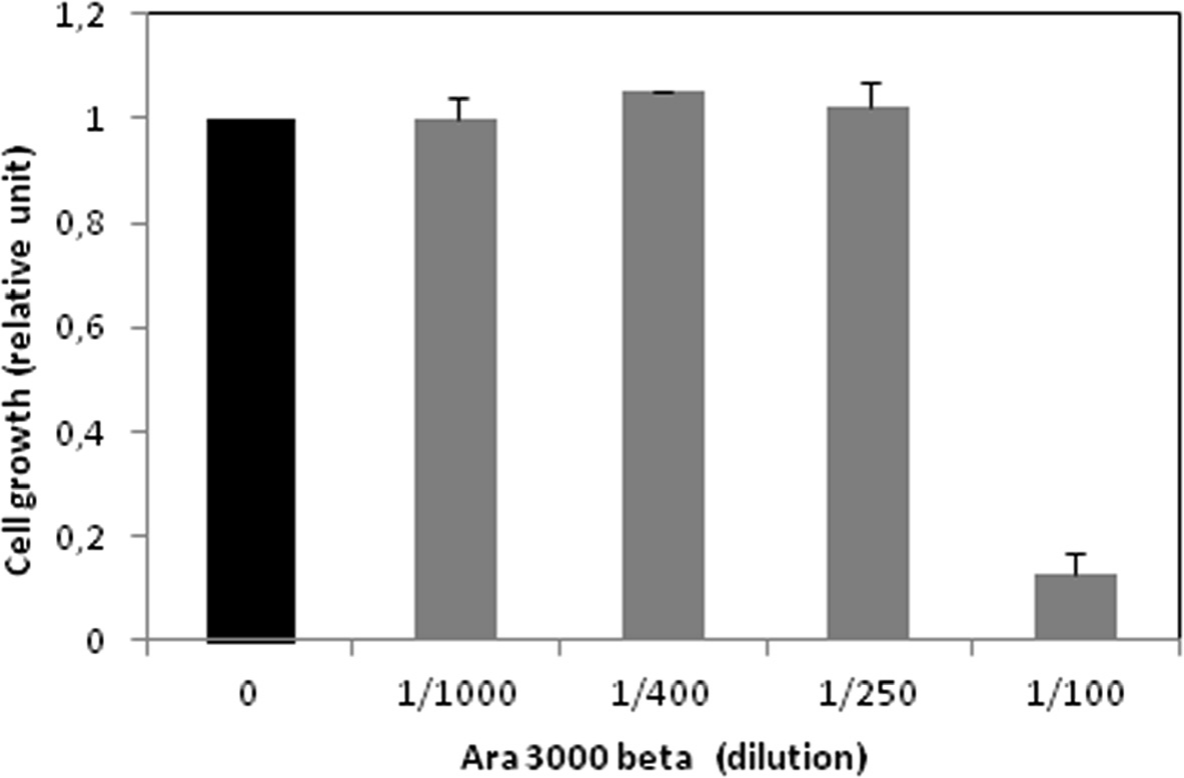
**Effect of Ara 3000 beta® on chondrocyte growth.** Human articular chondrocytes were cultured for 6–7 days. Then, cells were treated with increasing concentration of Ara 3000 beta® (1:1000 to 1: 100) for 48 h. At the end of incubation, cell growth was evaluated using Wst-1 assay. Values were normalized to OD from untreated cells.

In order to evaluate the putative effect of the fatty acid copolymer Ara 3000® on chondrocyte proliferation or toxicity, we evaluated the number of viable chondrocytes using WST-1 assay. Our analyses (Figure 1) showed that this copolymer of fatty acids (at dilution ≥ 1:250) had no effect on survival or proliferation of chondrocytes. However, we observed a strong toxicity at higher concentration.

### Effect of the copolymer of fatty acids on MMPs

**Figure 2:**
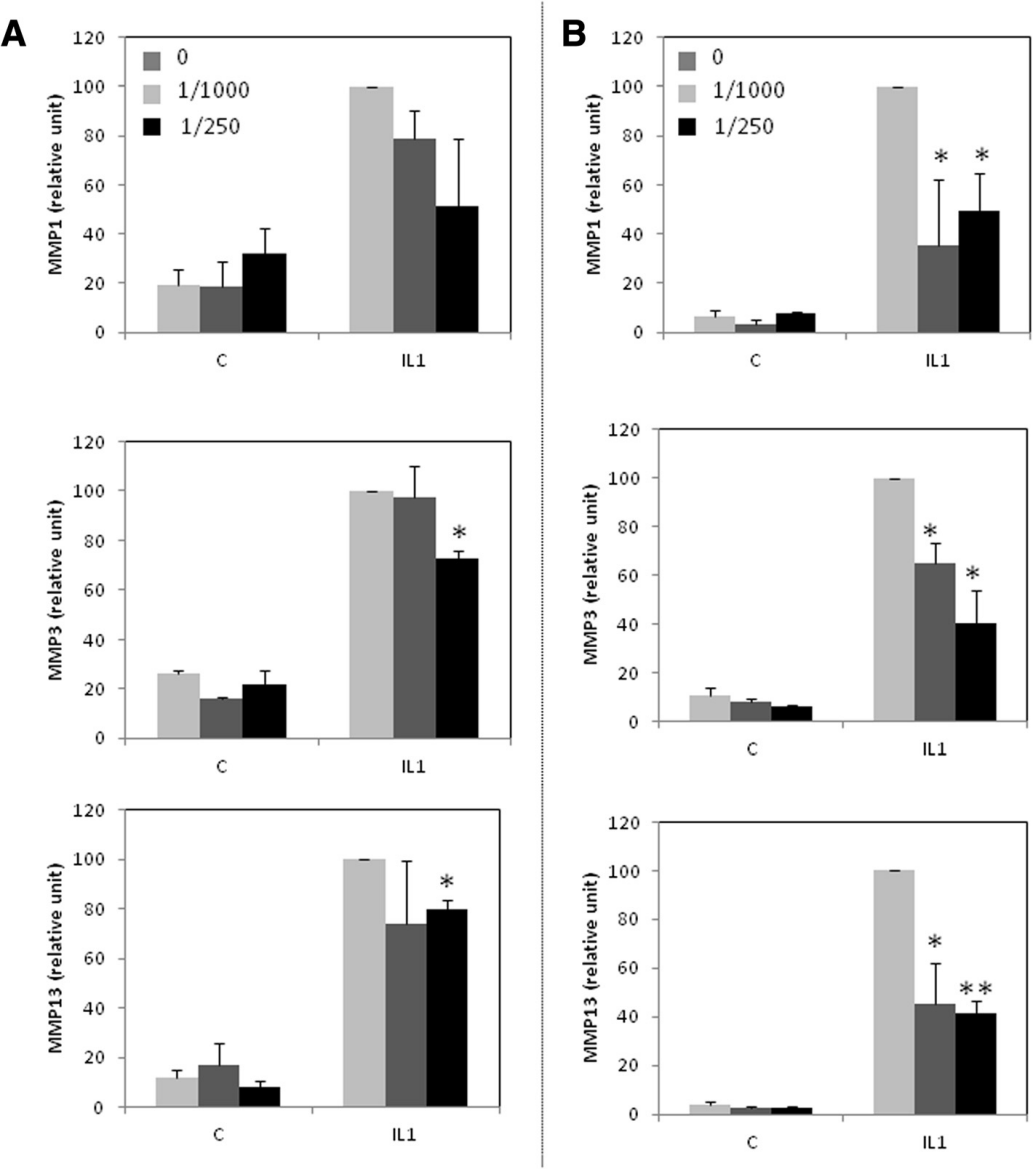
**Effect of Ara 3000 beta® on IL-1 induced MMP mRNA expression.** Human articular chondrocytes were cultured for 6–7 days. Then, they were incubated with IL-1β (1 ng/ml) (IL1) or vehicle (C) in the presence or not of Ara 3000 beta® (dilution 1:250 or 1:1000). At the end of the incubation, MMP mRNA level was evaluated by RT-PCR. **A**- Cells were co-treated with IL-1 and Ara 3000 beta® for 48 h. **B** - Cells were incubated with Ara 3000 beta® for 24 h. Then medium was changed and cells were cotreated with IL-1 and Ara 3000 beta® for 24 h.

We pursued this study by analyzing IL-1 induced-expression of MMP1, MMP3 and MMP13 (Figure 2). A 48 h-treatment with the copolymer of fatty acids clearly prevented IL-1ß-stimulation of MMP mRNA levels, particularly at 1:250. This effect was more significant when cells were pre-incubated with the copolymer of fatty acids for 24 h before stimulation by IL-1. That’s why we use this experimental condition for further study.

**Figure 3:**
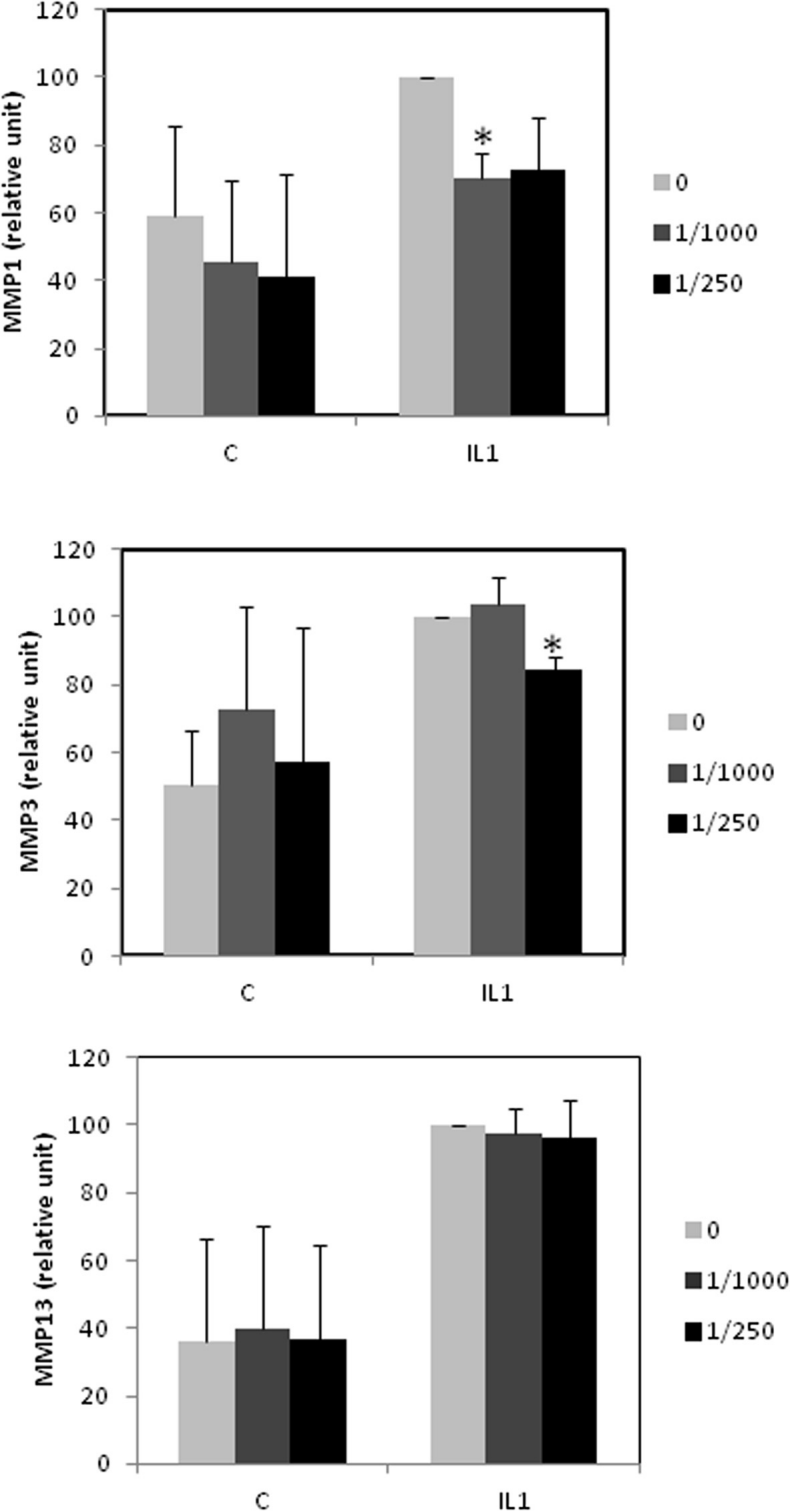
**Effect of Ara 3000 beta® on IL-1 induced MMP release.** Human articular chondrocytes were cultured for 6–7 days. At 80% confluency, cells were preincubated with Ara 3000 beta® (dilution 1:250 or 1:1000). The day after, medium was changed and cells were cotreated with IL-1 (IL1) or vehicle (C), and Ara 3000 beta® for 24 h. At the end of the incubation, release of MMPs was evaluated by ELISA. (Mean quantities of MMP released in untreated wells: MMP1:22 μg; MMP3:18 μg; MMP13:188 ng.)

We investigated the release of MMPs in medium by ELISA (Figure 3). Our experiments show that Ara 3000 beta® treatment reduces IL-1 induced-release of MMPs. However, this effect was statistically significant only for MMP1 at dilution 1:1000 and MMP3 at dilution 1:250.

### Effects of the copolymer of fatty acids on IL-1 induced-release of NO and PGE2

**Figure 4:**
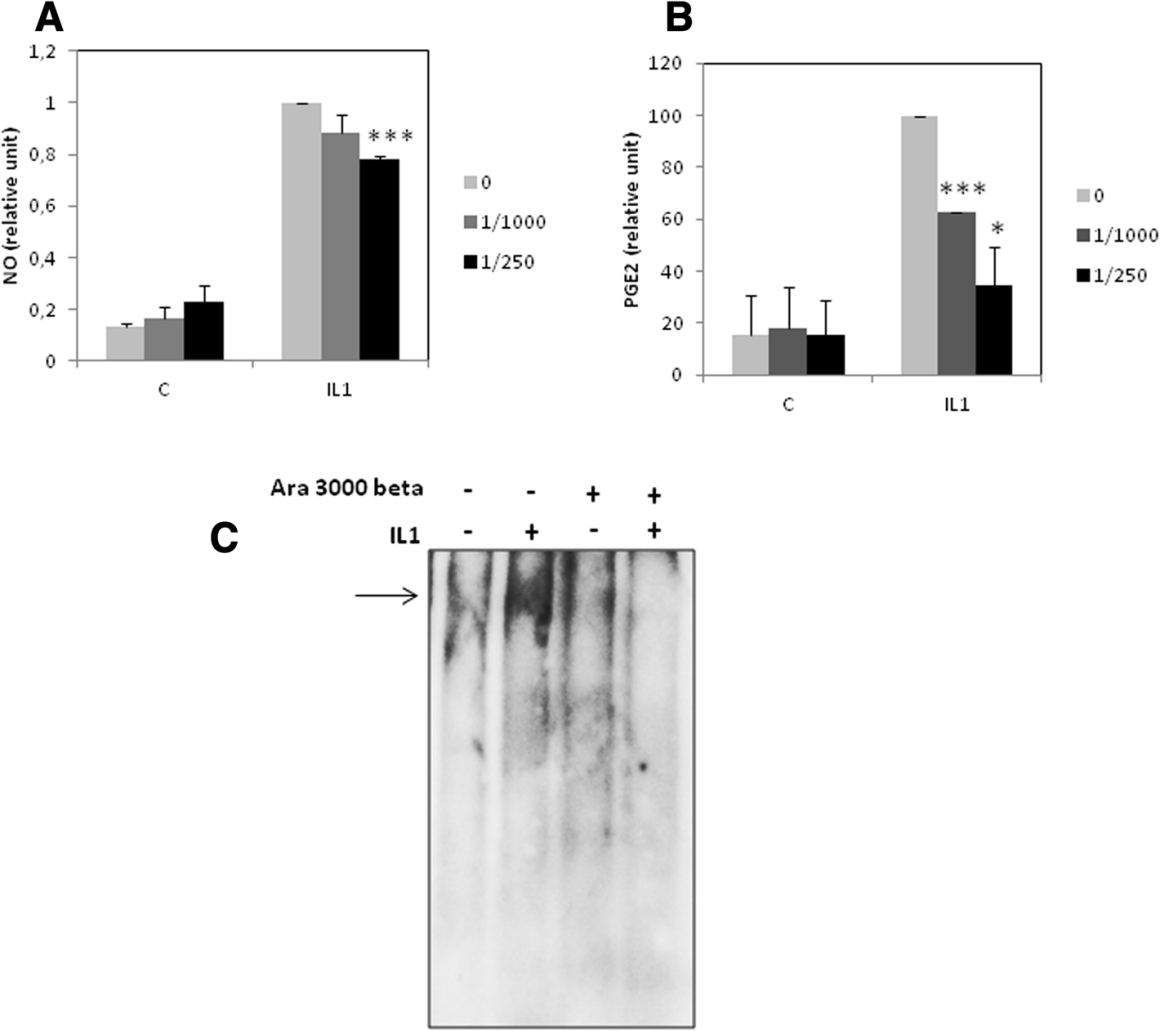
**Effect of Ara 3000 beta® on NO and PGE2 release, and NFκB DNA binding.** Chondrocytes were pre-incubated 24 hours with Ara 3000 beta® or vehicle, and then they were treated with IL-1β (IL1) or vehicle **(C)**, in the presence or not of Ara 3000 beta® for 24 h. **A**, **B**- At the end of treatment, mediums were collected, and NO and PGE2 levels were determined in medium by Griess assay **(A)** and ELISA **(B)**, respectively. Histograms represent the percentage of induction of NO or PGE2 in Ara 3000 beta® treated-cells normalized to cells treated with IL-1 without Ara 3000 beta®. **C**- Electrophoretic mobility shift assay (EMSA) were performed using NFkB consensus binding probe, incubated with nuclear extracts from chondrocytes treated as previously. Arrow indicates the band corresponding to NFkB binding.

We examined whether Ara 3000 beta® influenced the release of inflammatory molecules implicated in joint diseases, including NO and PGE2 (Figure 4A and B). By Griess assay or ELISA, we showed that this copolymer of fatty acids at dilution 1:1000 and 1:250 significantly reduced IL-1 induced-release of NO and PGE2.

### Effects of the copolymer of fatty acids on IL-1 induced-activation of NFkB

To determine whether these anti-inflammatory effects of Ara 3000 beta® could be associated with modulation of NF-kB activation, we evaluated DNA binding activity of NFκB by EMSA (Figure 4C). While IL-1β induced NFkB binding to DNA, this binding was impaired by Ara 3000 beta® co-treatment, confirming the anti-inflammatory effect of this fatty acid copolymer.

### Effects of a fatty acid copolymer in vivo arthritis model

**Figure 5:**
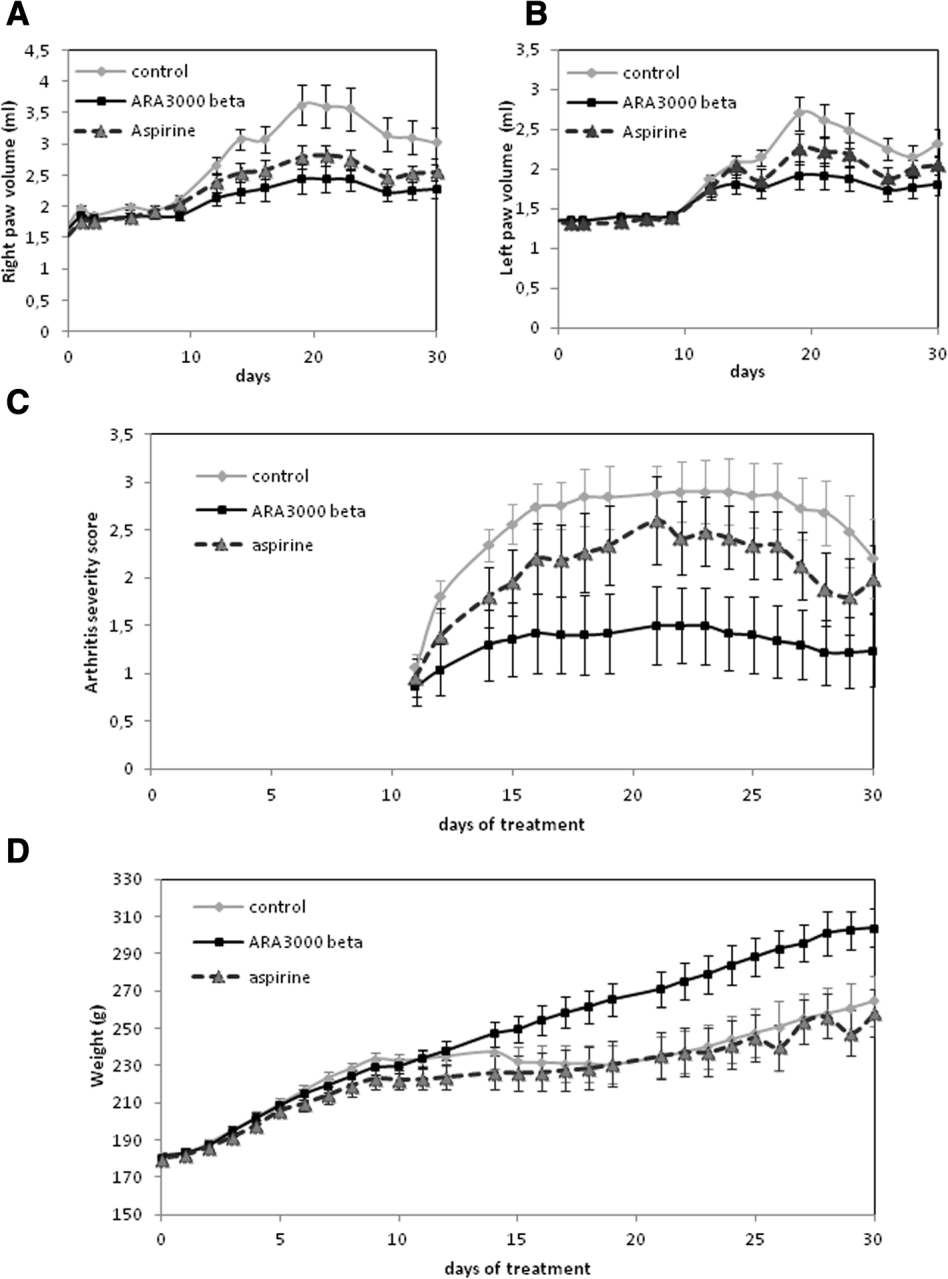
**Effect of Ara 3000 beta® in arthritis rat.** Arthritis was induced by injection of Freund Adjuvant. Then, rats were treated or not with Ara 3000 beta® or acetylsalicylic acid (aspirin), and edema **(A** **and** **B)**, arthritis severity score **(C)** and body weight **(D)** were determined each days during one month. Graphs represent mean values from 10 animals ± SEM.

Finally, we studied anti-inflammatory effect of the copolymer using adjuvant-induced arthritis (AIA) model in rats. Following injection of complete Freund’s adjuvant (CFA), we observed a significant inflammatory reaction in the right hind paw as soon as the first day, and the reaction progressively worsened during the following 20 days. An inflammatory response in the non-injected paw (left hind paw) was observed from the tenth day after the induction of AIA and increased gradually until day 21. Treatment of rats with acetylsalicylic acid did not significantly reduce inflammation. However, treatment of rats with Ara 3000 beta® reduced the development of edema in the right paw (injected with CFA) (p < 0.01 from day 12) and also in the left paw, which was not injected with CFA (p < 0.02 from day 16) (Figure 5). In addition, arthritis severity score from hind paws and tails shows a benefit of the copolymer of fatty acids (p < 0.05 from day 12). From day 14, animals without treatment develop a moderate and then severe inflammation (score > 2), whereas animals treated with the copolymer of fatty acids show only a weak inflammation (score < 1.5).

Concerning the weight of the animals, we observed an increased progressively until the 12th day, when the systemic manifestations of the disease began, at which time a slight weight loss occurred. Interestingly, Ara 3000 beta®-treated rats continued to grow until the end of experiments (day 30) (p < 0.03 from day 18).

## Discussion

This work investigated the therapeutic potential and anti-inflammatory effects of a copolymer of fatty acids composed of oleic acid, palmitic acid and stearic acid (Ara 3000 beta®) *in vitro* and *in vivo* in joint.

First, we measured MMP production, NO and PGE2 release, in a physiologically relevant *in vitro* model of inflammatory joint disease. IL-1β treatment was used at 1 ng/ml as in previous studies [16,17], concentration which powerfully stimulated inflammation and MMP production.

Our study shows that fatty acid polymer can modulate the expression of several genes implicated in the catabolism of extracellular matrix and its pathological dysfunction. We found that Ara 3000 beta® was capable of preventing IL-1-stimulated MMP-1, MMP3 and MMP13 expression and release. These data suggest a protective effect of fatty acid copolymer on cartilage degradation induced by interleukin-1β.

*In vitro*, reduction of MMP expression was more important when Ara 3000 beta® was used in pre-treatment, suggesting that this copolymer of fatty acids should be also use as preventive treatment. In this condition (24 h-pretreatment with Ara 3000 beta® followed of 24 h-treatment with Ara 3000 beta® in the presence of IL-1β), we show that this fatty acid strongly decreases the IL-1 induced-expression of MMPs at mRNA level (>40%). This effect was weaker when we evaluate MMP release in medium. However, it is noteworthy that 24 h-incubation with IL-1 induces a weak release of MMPs in medium, whereas it is sufficient to induce NFκB activation and a strong increase of MMP expression at mRNA level.

Interestingly, this reduction of MMP production by Ara 3000 beta® can be correlated with Bernadat’s observations which had showed, by proline release assay, that Ara 3000 beta® decreases protein degradation in articular chondrocytes [18].

In addition, we showed a clear anti-inflammatory effect of the copolymer of fatty acids, since it decreases both NFκB pathway activation and the release of inflammatory molecules such as NO and PGE2. This anti-inflammatory effect may prevent proinflammatory leukocyte adhesion receptor expression, and subsequent leukocyte-endothelial interaction. Therefore, the beneficial effect of the copolymer of fatty acids may be even more profound and longer lasting *in vivo*, where broader anti-inflammatory circuits can be engaged. This is in agreement with our *in vivo* observations which have showed using adjuvant-induced arthritis model, that Ara 3000 beta® has an anti-inflammatory effect on vascular and cellular phase of inflammation, characterized by the maintenance of rat growth and a reduction of edema and inflammation. This suggests that Ara 3000 beta® counteracts inflammation induced by IL-1 as shown by our *in vitro* data, but also that this copolymer of fatty acid may also reduce TNF-a and IL-6 effects, since these cytokines are strongly induce during adjuvant induced arthritis rat model [19]. However, other analysis would be required to confirm this hypothesis. Interestingly, we found a better effect of fatty acid polymer compared to acetylsalicylic acid. These data support the validity of clinical cases showing a benefit of Ara 3000 beta® against joint disease in dogs [14,20], and bring the proof of the anti-inflammatory effects of this fatty acid in articular chondrocytes.

The molecular mechanism by which Ara 3000 beta® exerts its anti-inflammatory effects is still unknown. However, several hypotheses may be proposed. This copolymer of non-esterified fatty acids (palmitic, oleic and stearic acids) might be incorporated into the phospholipids of cell membranes, or act via surface or intracellular “fatty acid receptors”, such as peroxisome proliferator activated receptors (PPARs) [21]. Therefore, changes in membrane phospholipid fatty acid composition may influence the function of cells. It may alter physical properties of the membrane by maintaining membrane order (“fluidity”) and modifying lipid raft formation. This could affect NFkB signaling pathway [21], leading reduction of MMP gene expression.

## Conclusions

In conclusion, this report shows that a copolymer of fatty acids such as Ara 3000 beta® reduces inflammation in joint. *In vitro*, it impairs IL-1 stimulated-MMP production and release, as well as the release of NO and PGE2 and the activation of NFκB. Furthermore, in vivo, it reduces edemas, erythemas and ankylosis in arthritic rats. Consequently, fatty acids are powerful anti-inflammatory molecules, and may be used to treat joint disease such as arthritis or osteoarthritis.
